# Impact of Microfluidization on the Emulsifying Properties of Zein-Based Emulsions: Influence of Diutan Gum Concentration

**DOI:** 10.3390/ma14133695

**Published:** 2021-07-01

**Authors:** Jenifer Santos, Luis A. Trujillo-Cayado, María del Águila Alcaide, María del Carmen Alfaro

**Affiliations:** 1 Departamento de Ingeniería Química, Escuela Politécnica Superior, Universidad de Sevilla, c/Virgen de África, 7, E41011 Sevilla, Spain; ltrujillo@us.es; 2 Departamento de Ingeniería Química, Facultad de Química, Universidad de Sevilla, c/Profesor García González, 1, E41012 Sevilla, Spain; mariadelaguilaag@hotmail.com

**Keywords:** microfluidization, zein, diutan gum, rheology

## Abstract

Microfluidization is a preparation method that can be used to obtain emulsions with submicron droplet sizes. The first objective of this study was to evaluate the influence of homogenization pressure and cycles on droplet sizes using response surface methodology. Secondly, the influence of the diutan gum concentration incorporated in the optimized emulsion on rheological properties, microstructure, and physical stability was investigated. Taking the response surface analysis into account, the emulsion processed at 20,000 psi after four cycles seemed to show the smallest Sauter diameter values. Hence, this emulsion was the starting point to incorporate diutan gum. Interestingly, the formation of a 3D network in the emulsion, observed by FESEM, was provoked by diutan gum. The emulsion formulated with 0.4 wt.% of diutan gum presented rheological gel properties and enhanced physical stability. This work highlights the importance of selecting optimized processing variables using the microfluidization technique and extends the knowledge of using diutan gum in combination with zein.

## 1. Introduction

Pickering emulsions, which can be defined as dispersed systems, stabilized by colloidal particles, present some advantages when compared with emulsions stabilized by surfactants. For example, Pickering emulsion stabilizers are able to perform coalescence reduction, droplet aggregation, and Ostwald ripening [1,2]. Natural Pickering stabilizers have no problems concerning biodegradability and compatibility in food and pharmaceutical products [3], in contrast to inorganic or synthetic Pickering stabilizers [4]. Because of this, food-grade colloidal particles have been used more and more as Pickering stabilizers, such as cellulose derivatives, modified starch, plant proteins, or cereal proteins. A cereal protein, zein, is considered a promising biopolymer of the 21st century [5]. Zein is the main storage protein of corn and is rich in prolamin. Its colloidal particles are very interesting, due to potential applications in the food industry. Nevertheless, prolamins present high hydrophobicity, which can be a problem in forming Pickering emulsions. Previously, different authors have researched how to adjust zein aggregation and its structure by modifying the pH of the solvent where zein is dissolved [6], as zein without surface modification cannot be used as an emulsifier close to its isoelectric point [7]. In addition, the stability of zein-based emulsions can be affected by particle concentration and ionic strength [8]. Microfluidization could also be an interesting method to modify the protein structure and its emulsifying properties [9,10]. On the other hand, some studies report that the interaction between prolamins and natural biopolymers reduce the hydrophobicity in order to form stable Pickering emulsions. For example, it has been proved that gum arabic, pectin, and xanthan gum can improve the hydrophilicity of zein, and their conjugates can act as effective emulsifiers [11,12,13]. However, there is no information available about the formation of a diutan gum–zein complex as an emulsifier. 

Diutan gum, obtained by *Sphingomonas* sp. ATCC53159 by aerobic fermentation, is a combination of β-D-glucose, β-D-glucuronic acid, and α-L-rhamnose units that form a biodegradable and biocompatible anionic biopolymer [14,15]. This little-known polysaccharide, which belongs to the sphingans group, possesses a molecular weight from 2.88 to 5.18 MDa. In addition, it has proved its stabilization role by reducing creaming in lemongrass nanoemulsions [16]. In addition, it is possible to modulate the viscoelasticity and viscosity of diutan gum solutions as a function of its concentration in order to enhance the physical stability of the emulsions developed [17]. However, there is no information available about this polysaccharide used in combination with a protein as an emulsifier.

Many types of methods can be used to prepare food emulsions: ultrasonication, rotor–stator, and high-pressure homogenizers and microfluidizers. The microfluidization technique that is based on passing a pre-emulsion through microchannels on the interaction chamber at a very high shear can obtain droplets in the submicron range [18]. The droplet breakup takes part due to the impact of two impinging jets. In this process, high turbulence and a tremendous shearing action are created. Consequently, this forces the flow stream to pass through the well-defined microchannels in the interaction chamber. Concerning interaction chambers, there are two different types: Type Y and Type Z [19]. The Type Y chamber possesses microchannels of 75 µm, while the Type Z has channels of 200 µm, with the Type Y chamber reaching up to 8 × 10^6^ s^−1^ of shear and the Type Z reaching up to 2 × 10^6^ s^−1^. Furthermore, it has been proved that the use of Y + Z chambers in series increases the capacity of the device to obtain not only smaller droplets but also narrower droplet size distributions [20,21].

Microfluidization presents some advantages over the traditional methods. For example, microfluidization can be applicable in industrial operations and provides a flexible control of the emulsion droplet size. In addition, it can produce fine emulsions from a wide range of materials such as vitamins, bioactive lipids, drugs, antioxidants, and flavors [22,23]. Compared to the other methods, microfluidization can obtain smaller and narrower droplet size distributions [23]. These facts can reduce some destabilization mechanisms such as creaming, flocculation, and coalescence, improving the shelf life of the emulsions developed [19].

The main goal of this study was to develop stable emulsions based on zein. Extensive research about the microfluidization method on the formation of zein-based emulsions was carried out. The effect of microfluidized processing parameters such as homogenization pressure and number of cycles on droplet size distributions were analyzed by response surface methodology. Finally, the t influence of the diutan gum concentration on rheological properties and physical stability of these food emulsions were evaluated. This study brings to light the importance of adjusting the microfluidization technique for specific emulsions such as zein-based emulsions and extends the knowledge of using diutan gum in combination with a protein as a stabilizer.

## 2. Materials and Methods

### 2.1. Materials

Zein protein and rosemary essential oil, used as a natural food preservative, were provided by Sigma Aldrich (San Luis, MO, USA). Sunflower oil containing 40 wt.% of oleic acid was bought in the local supermarket. The gum solutions were prepared using KELTROL^®^ diutan gum, donated by CP Kelco (Atlanta, GA, USA), with deionized water.

### 2.2. Microfluidization of Food Emulsions Formulated with Zein Protein

The formulation of the emulsions developed was as follows: 2.1 wt.% of zein, 40 wt.% of sunflower oil, and 0.25 wt.% of rosemary essential oil (natural food preservative) [24]. This formulation was previously optimized by Santos et al., 2020 [11]. In the first part of this research, the influence of homogenization pressure and cycles through a microfluidizer on droplet size distributions was studied. The continuous phase contained zein protein and deionized water. The pH was adjusted to 11.5 using a NaOH solution. Then, a coarse emulsion (batches of 250 g) was prepared, at room temperature, using a Silverson L5 M for 60 s at 8000 rpm. Finally, finer emulsions were homogenized using a Microfluidizer M110P (Microfluidics company, Westwood, MA, USA) at different processing parameters (see Table 1). The microfluidizer was used with a configuration of Y + Z and a refrigeration temperature of 20 °C.

The emulsion with optimized values of processing was used as a starting point for the addition of the biopolymer (diutan gum, DG) to form a protein–polysaccharide complex. A primary gum solution containing 1.6 wt.% of DG was prepared using an IKa-Visc MR-D1 homogenizer for 8 h at 500 rpm at 20 °C. Then, this solution was kept at 7 °C for at least 48 h for the complete hydration of the polysaccharide. The final emulsions were prepared by mixing the gum solution and the optimal emulsion using the Ikavisc MR-D1 at 500 rpm for 10 min. Final emulsions containing different DG concentrations were obtained (0.1, 0.2, 0.3, and 0.4 wt.%).

### 2.3. Experimental Design and Data Analysis

An experimental design and response surface methodology were used to analyze the relationship between the dependent variables (mean droplet diameter and span) and independent variables (number of cycles and homogenization pressure). The experimental design consisted of five levels and two factors, generating 5^2^ = 25 experiments (see Table 1). The experiments were carried out in duplicate, and the design contained three additional center points (corresponding to 3 cycles at 15,000 psi), therefore yielding 28 samples. All the data were analyzed with a one-way analysis of variance (ANOVA) at a 95% confidence level. For the model construction, terms with *p* > 0.05 were removed and the analysis was recalculated without these terms. All the experimental design and data analyses were performed using the Echip software (Experimentation by Design, Wilmington, DE, USA).

### 2.4. Droplet Size Analysis

Droplet size distributions of the emulsions developed were characterized by using a Malvern Mastersizer 2000 (laser diffraction technology) (Malvern Instruments, Malvern, UK). In order to characterize these distributions, the Sauter diameter and span value were calculated as follows:

(1) $$D_{3.2}=\overset{N}{\underset{i=1}{\sum }}\frac{n_{i}d_{i}^{3}}{n_{i}d_{i}^{2}}$$

(2) $$span=\frac{d_{90}-d_{10}}{d_{50}}\;$$

where *d_i_* is the droplet diameter, *N* is the total number of droplets, *n_i_* is the number of droplets having a diameter *d_i_*, and *d*_90_, *d*_50_, and *d*_10_ are the diameters at 90%, 50%, and 10% cumulative volume.

### 2.5. Rheological Characterization

Rheological tests were carried out using a controlled-stress rheometer MARS II (Thermo Fisher Scientific, Waltham, MA, USA) equipped with a serrated plate–plate (60 mm diameter; 1 mm gap) in order to avoid slip effects. Stress sweeps at 1 Hz were performed in order to obtain the linear viscoelastic range (LVR) of the different samples. Frequency sweeps were carried out from 20 to 0.05 rad/s at a stress in the LVR at 20 °C. In addition, flow curves were conducted using a multistep protocol of 3 min per point (20 points) at 20 °C.

### 2.6. Field Emission Scanning Electron Microscopy (FESEM)

The optimized emulsion containing 0.4 wt.% DG was observed by the FESEM technique. This sample was fixed using glutaraldehyde (4 wt.%, cacodylate 0.1 M) and osmium tetroxide (1 wt.%, cacodylate 0.1 M). Subsequently, it was dehydrated with ethanol and acetone. Then, the sample was dried using a critical point dryer (CPD; Leica EM CPD 300, Wetzlar, Germany). The conditions of the dehydration and the drying used were reported by Santos et al., 2020 [11].

### 2.7. Physical Stability

In order to evaluate the physical stability of the emulsions developed, the multiple light scattering technique was conducted. Measurements of backscattering (BS) were carried out at different times of aging to identify and characterize the destabilization processes that could take place in the emulsions. In addition, the Turbiscan stability index (TSI) was calculated to clearly compare the kinetics of the destabilization process (Equation (3)):

(3) $$TSI=\underset{j}{\sum }\left|scan_{ref\;}\left(h_{j}\right)-scan_{i}\left(h_{j}\right)\right|$$

where *scan_ref_* and *scan_i_* are the initial backscattering value and the backscattering value at a specific time, respectively, and *h_j_* is a specific height in the measuring cell.

## 3. Results and Discussion

### 3.1. Influence of Processing Parameters in Microfluidizer on Droplet Sizes

**Figure 1:**
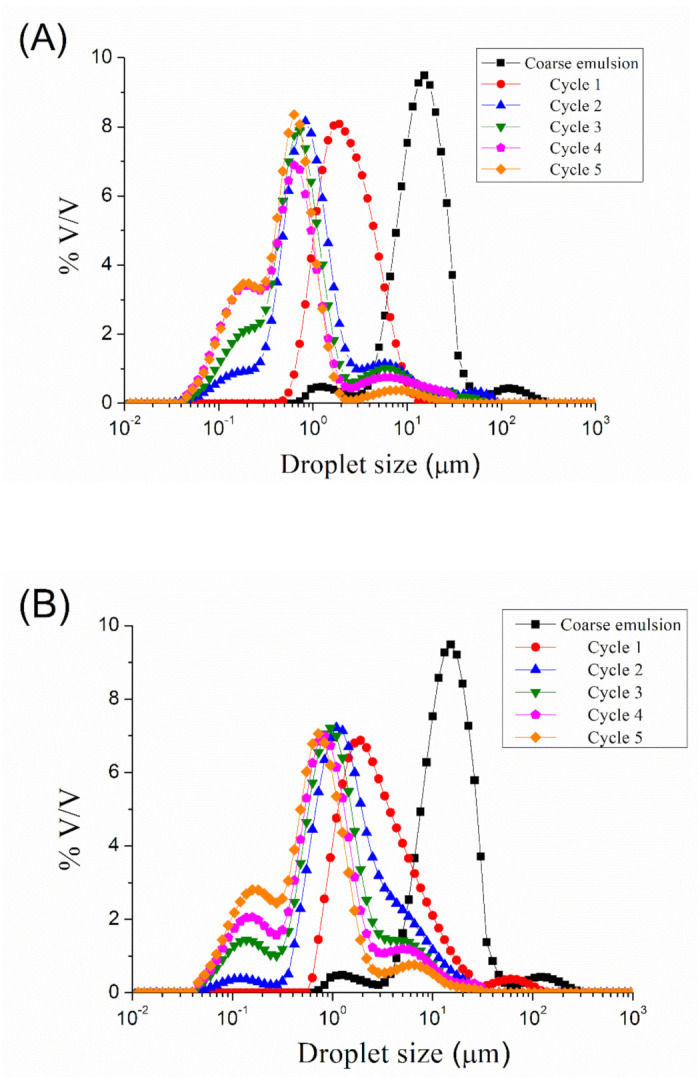

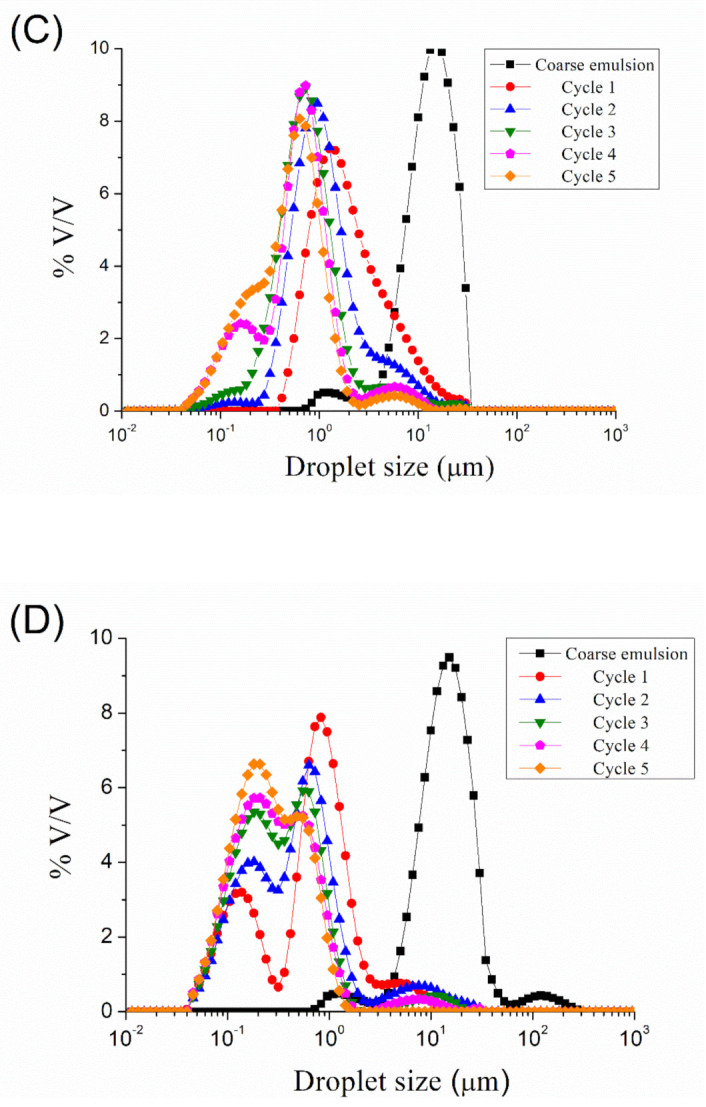

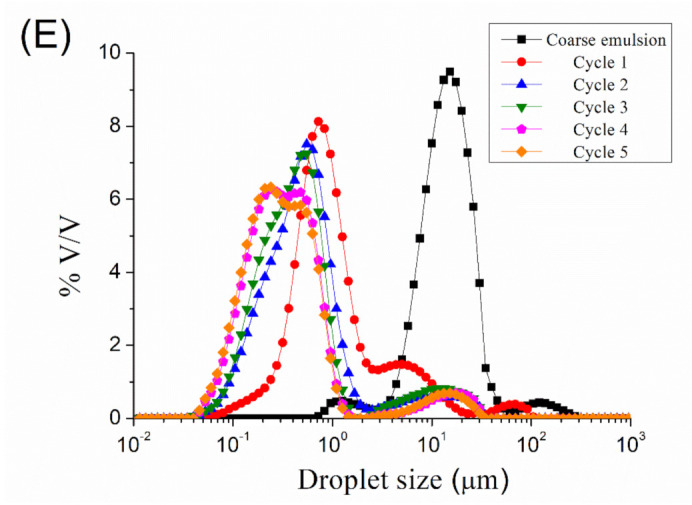
(**A**) Droplet size distributions for emulsions processed by microfluidization at 5000 psi as a function of the number of cycles. (**B**) Droplet size distributions for emulsions processed by microfluidization at 10,000 psi as a function of the number of cycles. (**C**) Droplet size distributions for emulsions processed by microfluidization at 15,000 psi as a function of the number of cycles. (**D**) Droplet size distributions for emulsions processed by microfluidization at 20,000 psi as a function of the number of cycles. (**E**) Droplet size distributions for emulsions processed by microfluidization at 25,000 psi as a function of the number of cycles.

Figure 1 show the droplet size distributions for emulsions processed by microfluidization at 5000, 10,000, 15,000, 20,000, and 25,000 psi, respectively, as a function of the number of cycles. A reduction in droplet size can be observed from coarse emulsion to emulsion processed with the microfluidizer, regardless of the homogenization pressure used. More cycles resulted in smaller droplet sizes. However, it seems that an increase in the number of cycles (cycles > 2) at 5000, 10,000, and 15,000 psi provokes an increase in the width of the peak, even in the appearance of a second peak. The microfluidization at 20,000 and 25,000 psi of the coarse emulsion led to a bimodal droplet size distribution at any number of cycles. The presence of a second peak at a larger droplet size than the main one is related to the recoalescence effect, due to over-processing emulsification.

**Figure 2:**
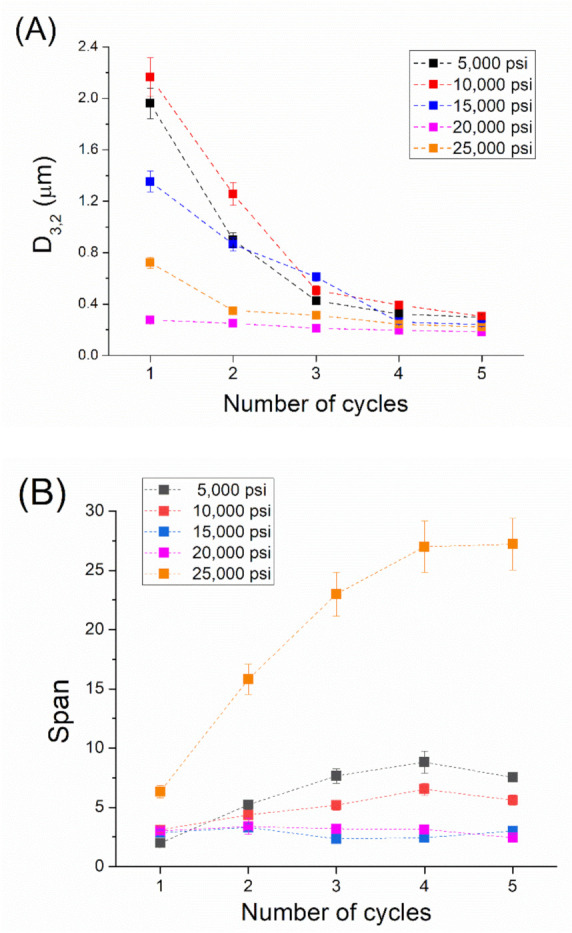
(**A**). Sauter mean diameter for the emulsions developed by microfluidization as a function of the number of cycles and the homogenization pressure. (**B**). Span values for the emulsions developed by microfluidization as a function of the number of cycles and the homogenization pressure.

In order to get a deeper insight into these results, Figure 2 is presented. Figure 2A illustrates the influence of the number of cycles on Sauter mean diameter (D_3,2_) as a function of the homogenization pressure. Generally, a reduction in D_3,2_ is observed with the number of cycles, regardless of the homogenization pressure. Interestingly, this figure brings to light the occurrence of submicron Sauter diameters at homogenization pressures higher than 15,000 psi. The relationship between submicron droplet diameter and longer physical stabilities is well known [25,26]. In addition, D_3,2_ obtained at 20,000 psi is smaller than that obtained at 25,000 psi, regardless of the pressure used. This fact could be due to the abovementioned over-processing.

On the other hand, the span values with the number of cycles as a function of the homogenization pressure are shown in Figure 2B. The most remarkable result in this figure is the increase in the number of span values with the number of cycles at 25,000 psi. This is not observed at other pressures. This unusual trend in span can be explained by the recoalescence effect due to over-processing.

**Figure 3:**
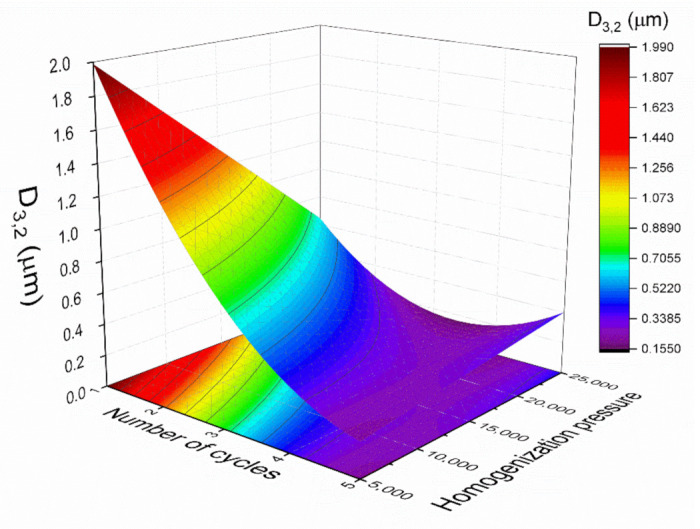
Response surface 3D plot of Sauter mean diameter (D_3,2_) as a function of the number of cycles and the homogenization pressure.

Response surface methodology was used to model the experiments statistically and optimize the parameters for emulsion development. Figure 3 indicates the relationship between the Sauter mean diameter (*D*_3,2_), with the homogenization pressure, and the number of cycles. The Sauter mean diameter was fitted with a quadratic function of the number of cycles (*C*) and the homogenization pressure (*P*):

(4) $$D_{3,2}=0.459-0.504\cdot C-0.289\cdot P+0.354\cdot C^{2}+0.382\cdot C\cdot P$$

The coefficient of determination (R^2^) value of 0.88 indicated a good correlation between the experimental results and the predicted responses. No significant lack of fit (Fcrit > Flof, with *p* = 0.05) was obtained, indicating that the model employed was adequate. Furthermore, in order to check the adequacy of the model, verification experiments were carried out with two different conditions: two cycles at 17,500 psi and four cycles at 7500 psi. The mean *D*_3,2_ of the emulsions developed at 17,500 psi and two cycles by triplicate was 0.42 ± 0.06 µm, which was close to the predicted value (0.41 ± 0.04 µm). In addition, the mean diameter value for the samples processed at 7500 psi and four cycles was 0.36 ± 0.01, while the predicted *D*_3,2_ value was 0.34 ± 0.02. Hence, the model was proved to be adequate. In this sense, the model predicted that *D*_3,2_ was sensitive to both studied variables, the number of cycles and homogenization pressure. In the present study, the increase in the number of cycles from one to four and the homogenization pressure from 5000 to 20,000 psi resulted in a decrease in the droplet sizes. However, when the homogenization pressure was increased from 20,000 to 25,000 psi, there was an increase in the droplet mean diameter. In addition, there are no significant differences between four and five cycles at 20,000 psi. Hence, the optimum processing variables were limited to four cycles and 20,000 psi. Taking all the results analysis into account, the emulsion processed at 20,000 psi after four cycles seems to show the smallest *D*_3,2_ values. For this reason, these conditions were fixed for the following tests.

### 3.2. Influence of Diutan Gum Concentration in Rheological Properties, Physical Stability, and Microstructure

**Figure 4:**
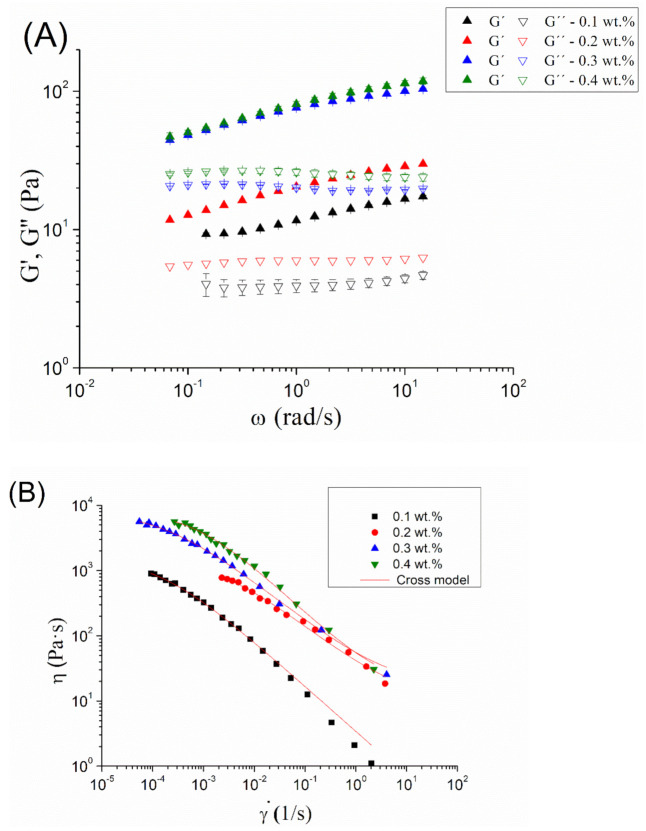
(**A**). Mechanical spectra for emulsions formulated with zein as a function of diutan gum concentration. (**B**). Flow curves for emulsions formulated with zein as a function of diutan gum concentration.

In order to enhance the physical stability of the optimized emulsion, a study of the influence of diutan gum concentration on viscoelastic properties was carried out (Figure 4A). Interestingly, G’ (elastic modulus) was higher than G’’ (viscous modulus) in all the frequency ranges studied at any diutan gum concentration. However, the differences of G’ and G’’ values were not large. These facts bring to light the existence of a weak gel-like behavior. The results obtained by Garcia et al., 2019 [17] showed a cross-over point in the mechanical spectra for the aqueous solutions of diutan gum. Hence, the appearance of zein and droplets have provoked the occurrence of a stronger network. Furthermore, there was an increase in G’ and G’’ with the diutan gum concentration, as expected.

Figure 4B presents the flow behavior for zein-based emulsions as a function of diutan gum concentration. All systems show a decrease in the apparent viscosity with shear rate, which is clear evidence of shear-thinning behavior. In addition, they are fitted to the well-known Cross model observed in Equation (5) (R^2^ > 0.98). The fitting parameters can be observed in Table 2.

(5) $$ƞ=\frac{{ƞ}_{0}-{ƞ}_{\infty }}{1+{\left(k\dot{\gamma }\right)}^{1-n\;}}$$

where ƞ is the viscosity, $\dot{\gamma \;}$ is the shear rate, ${ƞ}_{0}$ is the viscosity at very low shear rates, ${ƞ}_{\infty }\;$ is the viscosity at very high shear rates, k is the inverse of the critical shear rate, and *n* is the flow index.

There is an increase in zero-shear viscosity with diutan gum concentration, as expected. However, there is a jump in this parameter from 0.2 to 0.3 wt.% and a tendency to level off from 0.3 to 0.4 wt.%. This could be related to a depletion flocculation process. A depletion effect can provoke an attractive force between the oil droplets and form aggregates. This may increase the viscosity from 0.2 wt.% to 0.3 wt.%. Furthermore, flow index values did not present significant differences, showing similar shear-thinning degrees.

**Figure 5:**
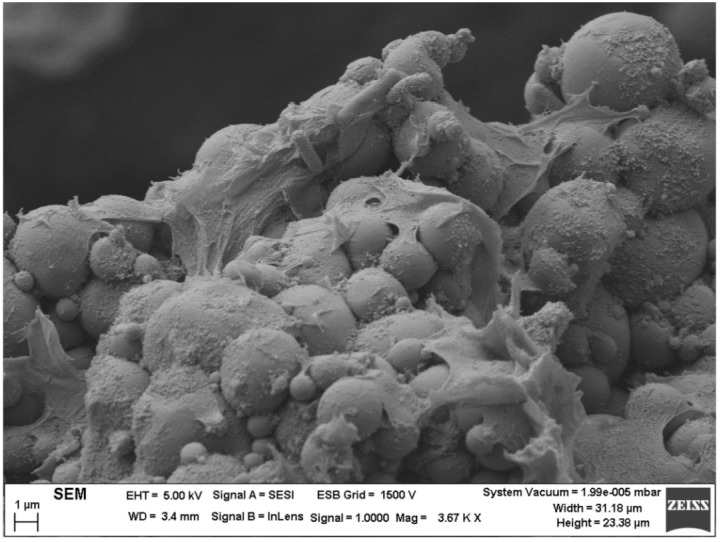
Representative cryo-SEM micrographs for the emulsion formulated with 0.4 wt.% of diutan gum.

In Figure 5, the microstructure created by the droplets, zein, and diutan gum is observable for emulsion containing 0.4 wt.% diutan gum. The droplets are very wide, as expected taking into account the laser diffraction results. In addition, the emulsion forms flocs, but these flocs are immobilized within the network of diutan macromolecules.

**Figure 6:**
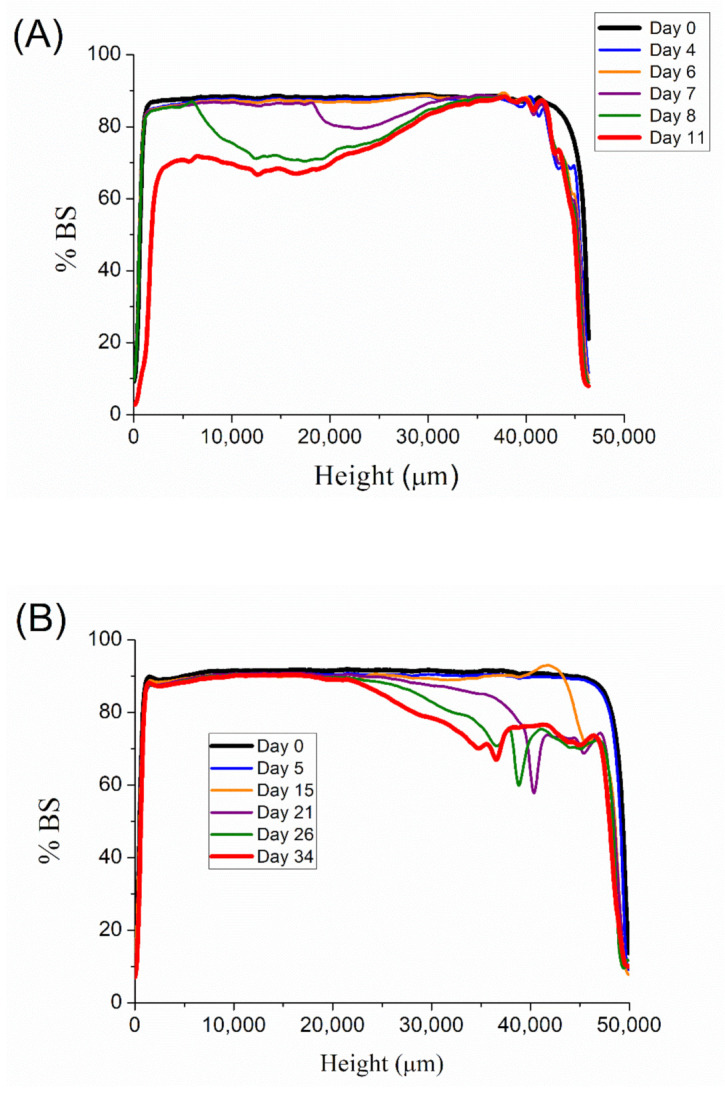

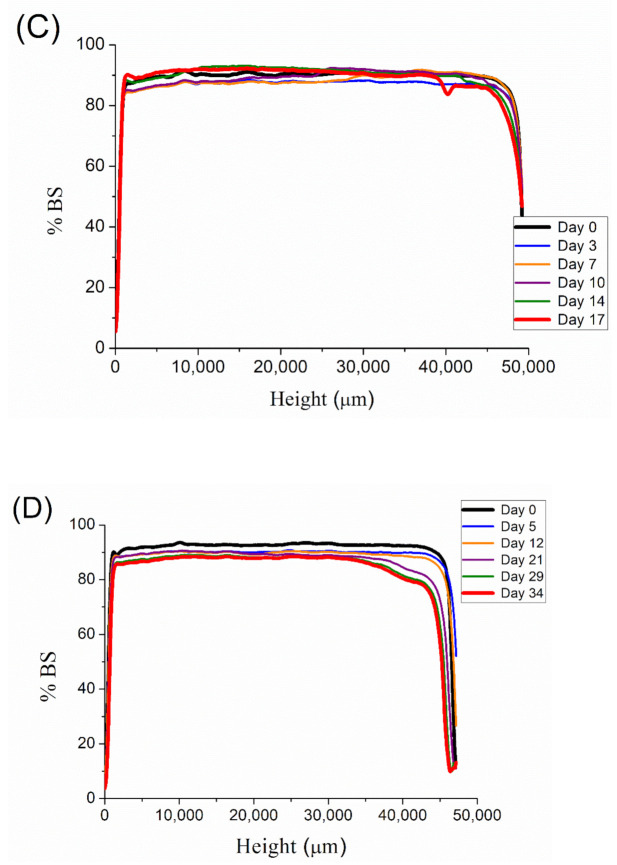
(**A**). Backscattering measurements with the height of the measuring cell as a function of aging time for emulsion containing 0.1 wt.% of diutan gum. (**B**). Backscattering measurements with the height of the measuring cell as a function of aging time for emulsion containing 0.2 wt.% of diutan gum. (**C**). Backscattering measurements with the height of the measuring cell as a function of aging time for emulsion containing 0.3 wt.% of diutan gum. (**D**). Backscattering measurements with the height of the measuring cell as a function of aging time for emulsion containing 0.4 wt.% of diutan gum.

Backscattering (BS) measurements were carried out in order to study the destabilization mechanism of these emulsions. Figure 6 illustrates the BS values with the height of the measuring cell as a function of aging time for emulsions with 0.1, 0.2, 0.3, and 0.4 wt.% of diutan gum, respectively. A decrease in BS in the low zone of the measuring cell is shown in Figure 6A. This fact is related to a clarification process in this zone, i.e., droplets in the low zone migrate to the upper zone leading to a creaming process. The possible solution to this destabilization process is the increase in the continuous phase viscosity. Therefore, emulsions with a higher content of biopolymer should be more stable. The backscattering values for 0.2 wt.% emulsion are shown in Figure 6B. There is a clear drop in BS in the upper zone of the measuring cell, which is related to a destabilization process called oiling-off. By contrast, 0.3 wt.% and 0.4 wt.% diutan gum emulsion do not show wide variations in BS values with aging time.

**Figure 7:**
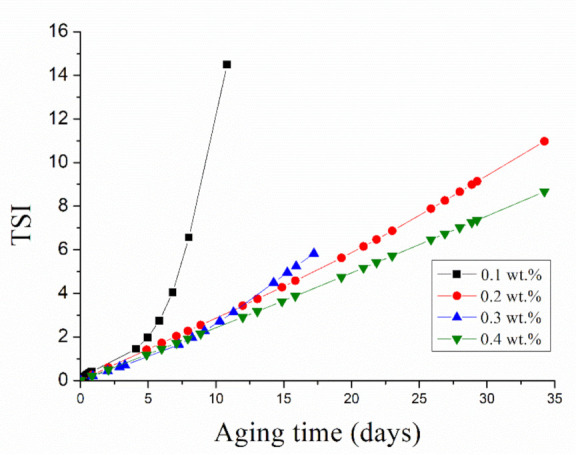
Influence of aging time on Turbiscan stability index as a function of diutan gum concentration for the emulsions developed.

In order to get a deeper insight into these results, Figure 7 is presented. This figure presents the Turbiscan stability index (TSI) values for the emulsions studied along with aging time. An increase in this value is directly related to a decrease in physical stability. Hence, 0.1 wt.% DG emulsion shows a marked increase in TSI in the first week after preparation. This is less and less acute with diutan gum concentration. The smallest variation of this parameter was obtained by the emulsion formulated with 0.4 wt.% of biopolymer. This result suggests that the strongest network (highest viscosity) is able to reduce the creaming process observed in the emulsion containing 0.1 wt.% of diutan gum.

## 4. Conclusions

Optimization of the microfluidization parameters in order to minimize the Sauter diameter and the span value of emulsions containing zein and sunflower oil was carried out. It has been proved that microfluidization provokes multimodal distributions regardless of homogenization pressure after two cycles. Surface response methodology was a powerful tool to obtain a clear result regarding the optimized emulsion (20,000 psi and four cycles).

Once the microfluidization process had been optimized, diutan gum was evaluated to enhance the physical stability of emulsions formulated with zein. It was proved that the combination of zein and diutan gum form a stronger network than diutan gum solutions. In addition, the increase in diutan gum concentration showed higher values of viscoelastic moduli. In addition, this network formed was observed by the FESEM technique. This microstructure is the reason for the enhanced physical stability of a zein–diutan gum complex containing more than 0.1 wt.% DG. This study has revealed the impact of microfluidization on emulsions formulated with proteins and has developed systems with potential applications to encapsulate active ingredients.

## Figures and Tables

**Table 1:** Processing variable conditions used for the present study.

Independent Variable	Value	Level
Number of Cycles (C)	1	−1
	2	−0.5
	3	0
	4	0.5
	5	1
Homogenization Pressure (P)	5000	−1
	10,000	−0.5
	15,000	0
	20,000	0.5
	25,000	1

**Table 2:** Fitting parameter values for the Cross model. The values were obtained by a non-linear regression analysis.

Diutan Gum Concentration (wt.%)	η_o_ (Pa·s)	η_∞_ (Pa·s)	k (s)	*n*
0.1	1598	0.0001	7382	0.31
0.2	1620	10	453	0.37
0.3	8286	14	5395	0.38
0.4	8321	15	1346	0.27

Standard deviation of the mean (2 replicates) for η_o_, η_∞_ < 8%; Standard deviation of the mean (2 replicates) for k < 10%; Standard deviation of the mean (2 replicates) for *n* < 10%.

## Data Availability

The data presented in this study are available on request from the corresponding author.
